# Large Scale Cortical Functional Networks Associated with Slow-Wave and Spindle-Burst-Related Spontaneous Activity

**DOI:** 10.3389/fncir.2016.00103

**Published:** 2016-12-21

**Authors:** David A. McVea, Timothy H. Murphy, Majid H. Mohajerani

**Affiliations:** ^1^Department of Psychiatry, University of British ColumbiaVancouver, BC, Canada; ^2^Brain Research Centre, University of British ColumbiaVancouver, BC, Canada; ^3^Canadian Center for Behavioural Neuroscience, University of LethbridgeLethbridge, AB, Canada

**Keywords:** cortex, development, barrel cortex, plasticity, voltage sensitive dye imaging

## Abstract

Cortical sensory systems are active with rich patterns of activity during sleep and under light anesthesia. Remarkably, this activity shares many characteristics with those present when the awake brain responds to sensory stimuli. We review two specific forms of such activity: slow-wave activity (SWA) in the adult brain and spindle bursts in developing brain. SWA is composed of 0.5–4 Hz resting potential fluctuations. Although these fluctuations synchronize wide regions of cortex, recent large-scale imaging has shown spatial details of their distribution that reflect underlying cortical structural projections and networks. These networks are regulated, as prior awake experiences alter both the spatial and temporal features of SWA in subsequent sleep. Activity patterns of the immature brain, however, are very different from those of the adult. SWA is absent, and the dominant pattern is spindle bursts, intermittent high frequency oscillations superimposed on slower depolarizations within sensory cortices. These bursts are driven by intrinsic brain activity, which act to generate peripheral inputs, for example via limb twitches. They are present within developing sensory cortex before they are mature enough to exhibit directed movements and respond to external stimuli. Like in the adult, these patterns resemble those evoked by sensory stimulation when awake. It is suggested that spindle-burst activity is generated purposefully by the developing nervous system as a proxy for true external stimuli. While the sleep-related functions of both slow-wave and spindle-burst activity may not be entirely clear, they reflect robust regulated phenomena which can engage select wide-spread cortical circuits. These circuits are similar to those activated during sensory processing and volitional events. We highlight these two patterns of brain activity because both are prominent and well-studied forms of spontaneous activity that will yield valuable insights into brain function in the coming years.

## Introduction

A key feature noted by early investigators of brain activity was that its pattern depends on behavioral state. The most dramatic shift in behavior occurs when falling asleep, when the brain becomes much less responsive to the external world and focuses inwards on an internal one. Sleep would suggest a brain at rest, although it remains active with a structured set of ongoing spontaneous activity patterns that do not rely on external sensory inputs. Less is known about this spontaneous activity compared to activity evoked by external stimulation given the challenges of recording and interpreting it. In contrast to events related to sensory inputs or motor outputs, ongoing spontaneous activity has no fixed point to which it can be referenced. Factors that influence it are less clear and thus harder to experimentally control. Most challenging, though, has been the lack of a clear and consistent framework through which to examine it (Raichle, 2011; Hutchison et al., 2013).

Converging lines of evidence show spontaneous brain activity during sleep reflects the communication of brain regions to process past experiences and mediate learning and memory in the adult brain (Wilson and McNaughton, 1994; Huber et al., 2006; Euston et al., 2007; Ji and Wilson, 2007; Rasch and Born, 2007; Han et al., 2008; Schwindel and McNaughton, 2011; Bermudez Contreras et al., 2013; Barnes and Wilson, 2014). In addition, unique forms of spontaneous activity are present during sleep in the immature brain. These activity patterns, like the adult brain activity discussed above, are not reflective of current sensory or motor processes involving the external world. Studying this activity has significant benefits in that its function can be more easily tested, by disrupting it and examining the subsequent deficits in the resulting adult brain. Doing so has revealed crucial roles for this early network activity in measurable processes such as the formation of ocular dominance columns in the visual thalamus (Torborg et al., 2005), refinement of corticospinal projections (Martin et al., 2007), maturation of spinal reflex circuits (Grillner, 2004), and modulating the machinery of synaptic release in the hippocampus circuit (Mohajerani et al., 2007). Furthermore, there is substantial overlap between the processes that underlie developmental plasticity and that plasticity which continues through life in response to learning or injury (Singer, 1995; Caleo and Maffei, 2002; Wolpaw, 2002; Carmichael, 2003; Uhlhaas et al., 2010), suggesting that lessons learned via the study of spontaneous brain activity during development are likely to prove useful in understanding the adult brain as well (Constantine-Paton et al., 1990; Murphy and Corbett, 2009; Ben-Ari et al., 2012).

In this review article, we discuss two prominent forms of spontaneous activity in sensory cortices during sleep or under light anesthesia, one found in adult brains and one in developing brains. First, we discuss recent findings related to the slow-wave activity (SWA), a 0.5–4 Hz oscillation in the sleeping adult brain which synchronizes sensory, motor and association cortices in non-REM (NREM) sleep (Contreras and Steriade, 1997). Second, we discuss spindle bursts, a pattern of intermittent fast oscillations at 5–25 Hz in the developing brain which play an important role in maturation of sensory systems (Khazipov and Luhmann, 2006; Blankenship and Feller, 2009; Blumberg et al., 2013; Cirelli and Tononi, 2015). In discussing how both patterns are generated and shaped by neural circuits, we hope to reinforce the concept that sleep-related activity within sensory systems and other regions of the brain are neither noise nor idle “holding-patterns”, but rather are actively formed and deployed for specific purposes.

## Slow Wave Activity

Since the earliest days of EEG recording, sleep was a major target of research (Adrian and Matthews, 1934; Berger, 1969). Researchers made rapid progress, identifying and categorizing major stages of sleep based on distinct brain activity as well as behavior (Hirshkowitz, 2000; Rasch and Born, 2013). This promoted a view of sleep related activity as a set of distinct and isolated rhythms. More recently, a unified view of brain oscillations has become prominent. In this view, different brain rhythms are seen as elements of a more global complex oscillation grouping slow and fast frequency events (Steriade, 2006).

In a series of three articles, Steriade et al. (1993a,b,c) described a slow ~1 Hz oscillation that would support this view by providing a link between rhythms generated in the thalamus and their synchronous projection to the cortex during NREM sleep. Intracellularly, this oscillation was seen as a relatively rapid switch in the values of the membrane potential between silent (hyperpolarizing) and active (depoarizing by 7–10 mV) state. The two states are referred to as UP and DOWN states and the >1 Hz alternation between them is generally referred to as the *slow oscillation* (Van Someren et al., 2011). Usually, membrane potential fluctuations around the Up state are of higher amplitude, whereas the Down state is relatively free of membrane fluctuations (Steriade et al., 1993b). Its role in grouping faster rhythms within the thalamus and cortex, including delta- and spindle activity, has given rise to some confusion as to its naming, as it cannot fully be separated from the faster rhythms. Generally, the *term* *slow oscillation* refers to the <1 Hz component, while the term *SWA* to represent the broader collection of these rhythms and their power within the 0.5–4 Hz range (Mascetti et al., 2011).

The finding that cells from multiple regions of the cortex, along with subcortical structures, were synchronized by a single rhythm brought the newly described SWA a good deal of attention. Soon after its description in anesthetized cats, it was shown during natural sleep in cats (Steriade et al., 1996; Amzica and Steriade, 1998) and in humans (Achermann and Borbély, 1997; Simon et al., 2000) and it has since been demonstrated in rodents (Cowan and Wilson, 1994; Petersen et al., 2003; Doi et al., 2007; Ruiz-Mejias et al., 2011). Subsequent investigations have shown it exists in nearly all sensory, motor and association areas of the cortex (Steriade et al., 1993c; Ferezou et al., 2006; Mohajerani et al., 2013) and synchronizes the membrane potential of cells in different functional regions far removed from each other (Destexhe et al., 1999; Volgushev et al., 2006; Dickson, 2010).

## Precise Distribution of Slow-Wave Activity within Cortical Sensory Systems

More recently, a more precise distribution of SWA within specific cortical regions has been demonstrated (Genzel et al., 2014). In fact, intracranial depth EEG from regions across human brain showed many slow-waves are restricted to small regions of cortex, or begin regionally before traveling to broader areas (Nir et al., 2011; Botella-Soler et al., 2012). Furthermore, SWA can reflect the functional architecture of the brain; following learning tasks, for example, SWA can increase or decrease within localized functional systems (Huber et al., 2004, 2006; Iwasaki et al., 2004; Han et al., 2008; Hanlon et al., 2009; Bermudez Contreras et al., 2013).

**Figure 1:**
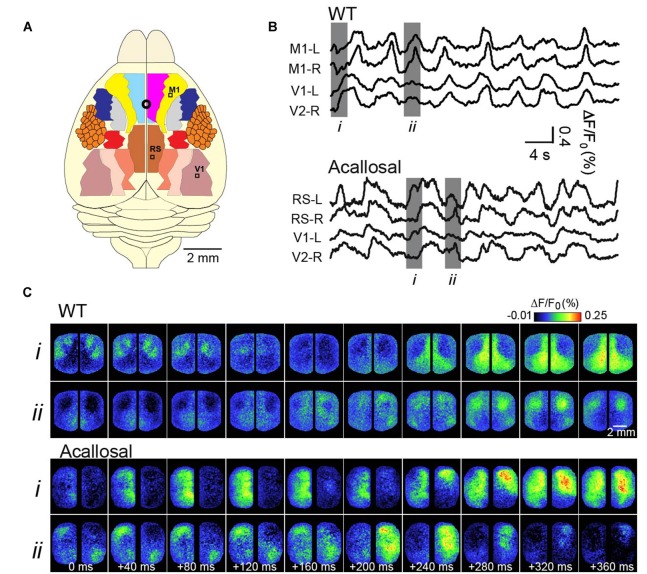
**Voltage-sensitive dye (VSD) imaging of slow-wave activity (SWA) in the adult anesthetized mouse. (A)** Schematic shows different cortical regions of the bilateral craniotomy preparation. Location of bregma is indicated by a white circle. **(B)** Example traces showing VSD signal from selected cortical regions in wild-type (WT) and acallosal I/LnJ mice that lack corpus callosum. M1, primary motor cortex; V1, primary visual cortex; RS, retrosplenial cortex **(C)**. Montages showing VSD images at time indicated in gray (*i, ii*) in **(B)**. Without the interhemispheric connections carried in the corpus callosum in acallosal mouse, spontaneous slow wave activity was less synchronized between hemispheres compared to the wild type animal. A, anterior; R, right; L, left; P, posterior. Republished with permission of Society of Neuroscience, (Mohajerani et al., 2010); permission conveyed through Copyright Clearance Center, Inc.

We and others, over the past years, have sought to expand these findings to determine precisely how SWA relates to the functional architecture of the brain, in turn giving further insights to its function (Kenet et al., 2003; Ferezou et al., 2006; Han et al., 2008; Mohajerani et al., 2010, 2013; Antic et al., 2016). Our strategy has been to use Voltage-sensitive dye (VSD) imaging of the rodent cortex, which has allowed collection of cortical activity at the mesoscale (tens of millimeters) spatial resolution and high temporal sampling, from very large regions of the cortex (Grinvald and Hildesheim, 2004). Examples of this activity, in a lightly anesthetized mouse, are shown in Figure 1 (adapted from Mohajerani et al., 2010). A crucial dependence on brain circuitry to shaping these patterns was demonstrated by the role of the corpus callosum in coordinating synchronous patterns of activity between hemispheres (Mohajerani et al., 2010).

**Figure 2:**
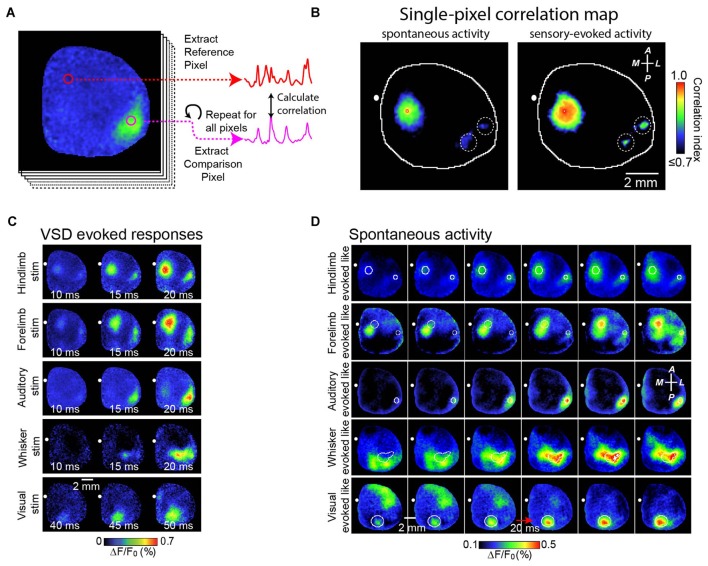
**Correlation maps show precise spatial layout of correlation of VSD signals from all pixels with VSD signal from each brain region.** To analyze patterns of spontaneous slow wave activity quantitatively, correlation maps based on a seed pixel have been shown to be an effective measurement. **(A)** Shows an example of this calculation. In this example, a sequence of voltage sensitive dye images is shown at the left. Using a seed pixel within an area of interest (reference pixel; red circle), one could obtain the correlation map by calculating its correlation to all other pixels within the imaged cortex. **(B)** Representative correlation maps from a seed pixel located within the primary representation of Hindlimb somatosensory area generated from VSD signals during 300 s of cortical spontaneous activity (left) or after sensory stimulation of the contralateral forelimb (right). There was a distant correlation at HLS2, as indicated by white dashed circles. CC, correlation coefficients (color coded). **(C)** Representative montages show examples of the VSD response to five different forms of sensory stimulation. **(D)** Representative montages showing cortical slow wave activity that resembles sensory-evoked cortical activity shown in **(C)**. A, anterior; R, right; L, left; P, posterior. Figure is adopted and modified with permission from Mohajerani et al. (2013).

We also studied the role of brain circuitry more precisely by examining how SWA is represented within functional cortical systems. Our strategy has been to collect long continuous stretches of cortical activity, and then to examine correlations of activity patterns between a functional region of interest and the remainder of the cortex (as shown in Figures 2A,B). In this way, subtle local patterns of activity emerge that are not necessarily distinguishable based on examination of raw signals. We have shown that, contrary to its early description as a global phenomenon that synchronizes broad regions of cortex, SWA activity has regional distribution which reflects the underlying axonal projections of cortex (Mohajerani et al., 2013; Vanni and Murphy, 2014). Figure 2A demonstrates this by showing a strong correlation of SWA within individual primary sensory regions or association regions. Indeed, we have shown that patterns of SWA can be temporally divided into combinations of activity within specific functional brain circuits (Figures 2C,D; Mohajerani et al., 2013).

We believe these results fit within a growing recognition that oscillations of SWA tap into functional networks of cortical cells (Castro-Alamancos, 2009; Harris and Thiele, 2011; Luczak and Maclean, 2012). This has been particularly well studied within sensory systems of the cortex. During the transition to UP states, the spatio-temporal pattern of spikes within a local network of cells is highly stereotyped. For example, the pattern of cell activation during UP states generated via thalamic simulation in slices (MacLean et al., 2005) or sensory stimulation *in vivo* (Luczak et al., 2009) is very similar to that of spontaneous UP states. On a larger scale of network also, slow-wave oscillations reflect functional connections with the visual (Kenet et al., 2003; Han et al., 2008; Mohajerani et al., 2013), somatosensory (Ferezou et al., 2006; Mohajerani et al., 2013) and auditory (Farley and Noreña, 2013; Mohajerani et al., 2013) cortices.

## Function of Slow-Wave Activity

These specific spatial patterns of SWA suggest it may be related to known sleep roles in learning and memory formation and consolidation. These roles come from studies which show sleep results in enhanced memory retention (Barrett and Ekstrand, 1972; Fowler et al., 1973; Plihal and Born, 1997; Walker et al., 2002; Tucker et al., 2006; Gais et al., 2008), by showing that performance on memory tasks after sleep is proportional to the amount of NREM sleep (Stickgold et al., 2000a,b), and that enhancing SWA via external electrical stimulation improves subsequent performance on memory tasks (Marshall et al., 2006; Binder et al., 2014). In addition, SWA is increased during sleep following social challenges (Meerlo et al., 2001), verbal learning tasks (Mölle et al., 2004), procedural motor learning tasks (Huber et al., 2004; Hanlon et al., 2009), or exploration (Vyazovskiy et al., 2005), but decreased following sensory deprivation (Iwasaki et al., 2004; Huber et al., 2006).

We believe the effects of sleep and SWA on memory formation are consistent with an emerging consensus of SWA having an important role in synaptic remodeling, a molecular correlate of learning. There are two main models of what sleep-related synaptic remodeling represents: replay of neural patterns associated with learning to promote synaptic plasticity (Sutherland and McNaughton, 2000; Schwindel and McNaughton, 2011); and down-scaling of new synapses created during normal waking or learning, to maintain a consistent overall excitatory balance in the brain (Tononi and Cirelli, 2006; Diekelmann and Born, 2010). The first model, known as active system reconsolidation, rests on an idea advanced by Marr (1970), namely that memories are encoded in short-term representations during waking, before being permanently encoded during sleep, when the brain is free of ongoing processing needs. In particular, it posits that memories are encoded temporarily in the hippocampus during exploratory activity, and then being “replayed” and transmitted to the cortex during NREM sleep via fast hippocampal sharp wave ripples (SPW-R), all under the synchronizing action of the slow oscillation (Buzsáki, 1996, 2015; Mölle and Born, 2011). Evidence for this theory includes: (a) NREM sleep primarily (but not exclusively, for example see Huber et al., 2004) benefits hippocampal dependent declarative memory consolidation (Stickgold and Walker, 2007); (b) recordings showing that cortical and hippocampal sequences of neuronal firing during waking are replayed together during NREM sleep (Wilson and McNaughton, 1994; Qin et al., 1997; Ji and Wilson, 2007) but not during REM sleep (Siapas and Wilson, 1998; Kudrimoti et al., 1999; Wierzynski et al., 2009); (c) hippocampal SPW-R are present at the transition between cortical UP and DOWN states of the slow oscillation (Battaglia et al., 2004; Mölle et al., 2006) and are temporally linked with both spindles and delta waves (Sirota et al., 2003; Johnson et al., 2010); and (d) selectively suppressing hippocampal SPW-R reduces sleep-related improvements in spatial memory (Girardeau et al., 2009).

The second model, that of synaptic homeostasis, comes from the observation that while synaptic potentiation and formation may be an effective mechanism for learning-related plasticity in the brain, it must be balanced by equivalent reductions in synaptic strength to avoid excess excitation (Miller and Mackay, 1994; Turrigiano, 2008; Tononi and Cirelli, 2014). Unfettered increases in synaptic strength and number also place energy and size burdens on the brain (Howarth et al., 2012). Rather than balancing increasing and decreasing of synaptic strengths in parallel, the synaptic downscaling theory of sleep suggest that synaptic potential proceeds mostly unhindered during waking before being selectively downscaled during sleep (Tononi and Cirelli, 2001, 2014).

Evidence is accumulating rapidly in support of this theory, and includes the following: (a) genes associated with synaptic potentiation (e.g., Arc and brain-derived neurotrophic factor (BDNF) are decreased during sleep (Cirelli and Tononi, 2000), while those associated with synaptic depression (e.g., Calcium/Calmodulin Dependent Protein Kinase IV (CAMK4), calcineurin) are increased (Cirelli et al., 2004); (b) cortical excitability as measured via frequency of spontaneous mini-excitatory post-synaptic potentials (Liu et al., 2010), spiking rates (Vyazovskiy et al., 2009), size of cortical evoked potentials (Hulse et al., 2011), or responsiveness to transcranial magnetic stimulation (Huber et al., 2013), increases with time awake and decreases following sleep; (c) direct visualization of synapses in both flies (Bushey et al., 2011) and mice (Maret et al., 2011) show that waking is associated with increasing numbers of synaptic spines, while sleeping is associated with a decrease; and (d) artificially enhancing synaptic potentiation by infusion of BDNF enhances subsequent SWA, but not REM, sleep activity in a region restricted to the infusion (Faraguna et al., 2008).

As mentioned earlier in this review article, activity-dependent synaptic potentiation is a major factor in our understanding of learning and memory. Active-system consolidation, with its focus on coordinated replay of salient sequences of neural activity, might be expected to promote synaptic potentiation via Hebbian plasticity by repeatedly activation of pairs of neurons. This would be in keeping with the observed benefits of sleep on memory, discussed earlier in this section. On the other hand, synaptic homeostasis emphasizes synaptic weakening and an elimination of non-relevant potential accrued during waking. Can these opposite effects be reconciled?

In fact, neither model of the role of SWA suggests that it contributes directly to synaptic potentiation. It has been known for some time that LTP is poorly induced during SWA (Leonard et al., 1987; Bramham and Srebro, 1989) and genes associated with LTP are down-regulated during SWA (Thiel et al., 1994; Jones et al., 2001). Thus, instead of triggering LTP directly, replayed activity during SWA may “tag” particularly salient synaptic changes, preserving them on a background of overall synaptic down-regulation (Ribeiro et al., 2004). Although the molecular underpinnings of this “tagging” are not clear, they may include persistently elevated calcium levels in selected cells (Rosanova and Ulrich, 2005; Diekelmann and Born, 2010). Alternatively, induction of some plasticity related genes, which respond strongly to pulsatile intracellular oscillation of calcium levels at SWS frequencies (Frey et al., 1993; Abel et al., 1997) could contribute (Sejnowski and Destexhe, 2000).

We believe recent results showing SWA reflects precise sensory circuits are consistent with both models. Important learning related changes can occur within very local cortical circuits (Komiyama et al., 2010; Fu et al., 2012) and local SWA within these circuits could facilitate this process, perhaps by permitting synchronized faster oscillation within components of the circuit (Yang et al., 2014). It bears noting that spontaneous activity within functional circuits may not have any particularly important function, but rather may simply reflect synaptic connectivity (Luczak and Maclean, 2012). However, localized changes in SWA that follow a specific learning task or sensory change suggest otherwise (Bermudez Contreras et al., 2013).

## Spindle Bursts—A Key Pattern of Spontaneous Activity in the Developing Brain

In the preceding section, we discussed patterns of activity in sensory systems of the brain when it is not required to be purposefully interacting with the environment because of sleep. During early life, the brain is also *less-able* to purposefully interact with the environment, as most sensory systems are not matured (Himwich, 1970; Jewett and Romano, 1972; Krug et al., 2001; Petersson et al., 2003; Colonnese et al., 2010), yet these systems display a variety of patterns of neural activity (Feller, 1999; Blankenship and Feller, 2009). There is a great deal of interest in this activity because it has been recognized to play essential roles in the normal development of the nervous system, acting in concert with genetically driven, molecular determinants of development (Goodman and Shatz, 1993; Tessier-Lavigne and Goodman, 1996; Chilton, 2006) to ensure formation of functional neural circuits (Pallas, 2001; Hanganu-Opatz, 2010; Blumberg, 2015).

A striking feature of the earliest recordable brain activity at approximately 24 weeks post-conception in humans, equivalent to approximately the time of birth in rats, is the lack of state-dependent differentiation. It does not display the defined patterns of continuous cortical activity that represent a particular behavioral state such as sleep or waking in the adult. Instead, the EEG consists mainly of bursts of high-amplitude undifferentiated waves, separated by periods of silence (Deza and Eidelberg, 1967; Anderson et al., 1985; Selton et al., 2000; Engel, 2008). Much of this early work on this pattern was spearheaded by French neonatologists and pediatric neurologists, led by Collette Dreyfus-Brisac, and the patterns are occasionally still referred to by their original French name, trace discontinue (discontinuous trace; Dreyfus-Brisac, 1978).

The absence of robust state-dependent patterns of EEG activity in early life does not mean that such states do not exist (Seelke and Blumberg, 2010). Some of the clearest transitions between states occur with regards to movements. As early as 10 weeks gestation in the human (Van Dongen and Goudie, 1980), and P2 (postnatal day 2) in the rat (Gramsbergen et al., 1970), alternating periods of quiescence and atonia, quiescence with general atonia but small multi-limb twitches, and large movements involving the whole body are seen (Corner, 1977; Robinson et al., 2000; Blumberg et al., 2005). These states are termed quiet sleep, active sleep and waking, respectively, and a consistent cycle of wake, quiet sleep, then active sleep can be seen in rats as young as P2 (Karlsson et al., 2004).

An important developmental milestone occurs at 28 weeks post-conception, when the human EEG remains discontinuous, but a prominent pattern of short 5–25 Hz bursts super-imposed on a slower 0.5–2 Hz background emerges during active sleep. The dominance of this pattern is illustrated by its many descriptions in the early literature (Khazipov and Luhmann, 2006), including “spindle-shaped bursts of fast waves” (Ellingson, 1958), “spindle-like fast rhythms” (Watanabe and Iwase, 2008), and “fast activity” (Goldie et al., 1971). These are now referred to *delta brushes* (8–28 Hz) in the clinical setting (André et al., 2010) and usually *spindle bursts* in the research setting. Khazipov et al. (2004) were the first to demonstrate that spindles bursts are linked to peripheral inputs. Recording from the hind- and forelimb somatosensory cortex of rats during the first week of life, they found that spontaneous limb twitches, as well as limb stimulation, were followed 100–200 ms later by spindle bursts.

**Figure 3:**
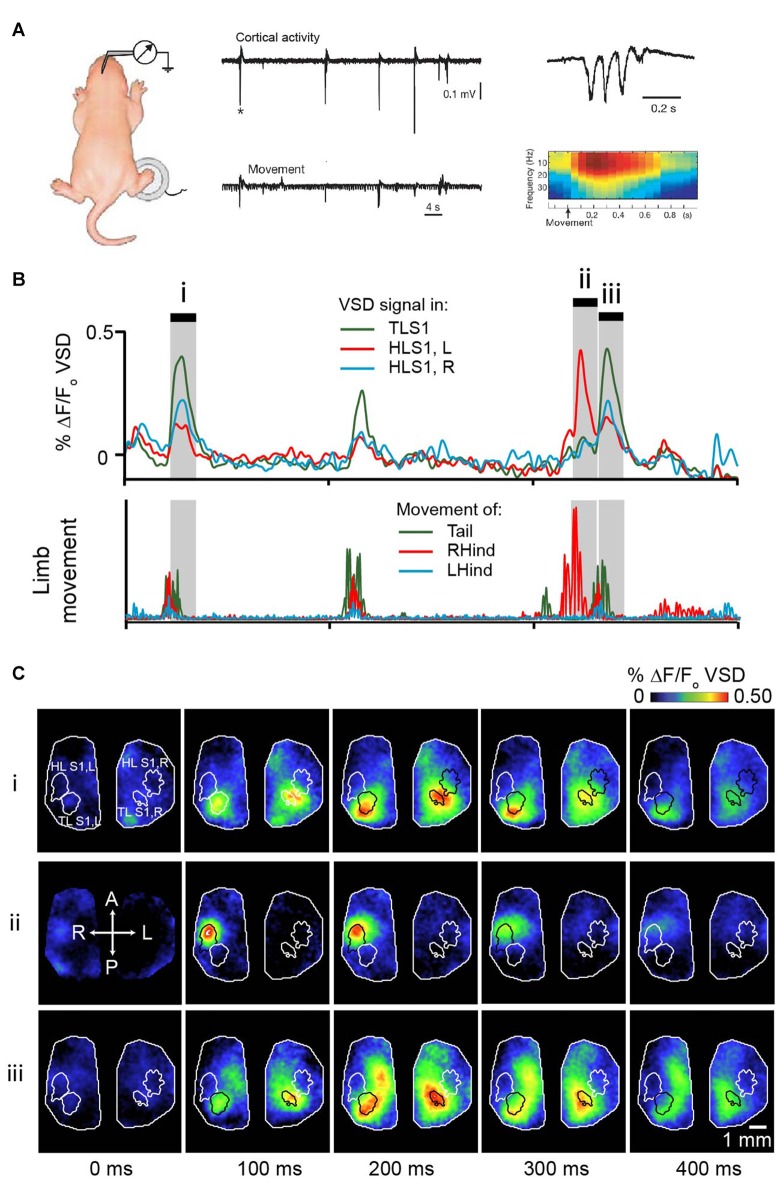
**Movement-triggered spindle bursts in primary somatosensory cortex of the newborn rat. (A)** Simultaneous recordings of the intracortical field potential in left hindlimb somatosensory cortex (top trace) of a 2 days old (P2) behaving rat. The bottom trace is the actual right-hindlimb movements. Note that the cortical spindle bursts are correlated with the movements. Right, spindle-burst marked by asterisk is shown on an expanded timescale (top) and below is corresponding time-frequency analysis. Figure is adapted and modified from Khazipov et al. (2004), used with permission from Nature through RightsLink. **(B)** Bursts of activity in sensory cortices of a 5 days old rat pup following movements of the limbs or tail. Pup is lightly anesthetized with isoflurane to approximate active sleep. Top panel shows VSD signal from tail sensory cortex, and left and right hindlimb cortex. These brain regions are outlined in **(C)**. Bottom panel concurrent signal of limb and tail movement collected with high speed camera. **(C)** Montages showing cortical activity every 100 ms in shaded gray regions highlighted in **(B)**. Republished with permission of Society of Neuroscience, (McVea et al., 2012); permission conveyed through Copyright Clearance Center, Inc.

Analogous results were also shown in preterm human infants; the clinically well-known delta brush pattern in the somatosensory cortex follows spontaneous hand and foot movements (Milh et al., 2006). The delta (0.5–2 Hz) and spindle burst (5–25 Hz) differ in their source, the former being mostly representative of cutaneous inputs and the latter, representative of proprioceptive inputs (Marcano-Reik and Blumberg, 2008). Spindle bursts remain generally localized to the corresponding sensory system. Twitches in the fore- and hindlimb give rise to spindle bursts in the fore- or hindlimb sensory cortex, respectively (Khazipov et al., 2004; Figure 3A). Using voltage sensitive dye (VSD) imaging in cortex of rat pups, we have shown that highly specific patterns of activation across the cortex follow hindlimb or tail twitches (McVea et al., 2012; Figures 3B,C). Importantly, this characteristic generalizes across the cortex; spontaneous retinal activity (a prominent feature of the visual system prior to eye opening (Feller, 1999; Feller and Scanziani, 2005; Firth et al., 2005; Torborg et al., 2005), or optic nerve stimulation gives rise to spindle bursts in the visual cortex (Hanganu et al., 2006), whisker twitches or whisker stimulation give rise to spindle bursts in the whisker somatosensory cortex (Minlebaev et al., 2007; Yang et al., 2009; An et al., 2014). In all cases, disrupting ascending sensory inputs (via spinal transection, whisker pad anesthesia, or retinal inactivation) decreases but not completely stopped the incidence of spindle bursts significantly in the corresponding cortex.

## Purpose of Spindle Bursts

Although our understanding of the function of cortical activity in sleep during infancy remains limited, there are clues that spindle bursts play important developmental roles. Limb twitches during development have a clear instructive role to the nervous system. This was shown directly by manipulating the sensory feedback that arrives following a spontaneous twitch in 2-week old rat pups (Pearson et al., 1999). When an air puff was used to artificially stimulate the side of the tail opposite to the movement direction (a reversal of what would occur normally), the rat later made more errors in withdrawing from stimuli applied to the tail. The authors concluded that the sensory feedback provided by the twitch is used to aid in the maturation of pain-withdrawal reflexes. Nearly five decades ago, Roffwarg et al. (1966), suggested that high levels of active or REM sleep in early life could serve to provide the developing cortex with activity when little is available from the outside world. Could the spindle bursts that accompany the twitches of active sleep be a cortical manifestation of this hypothesis, and have an instructive role similar to that seen in the pain-withdrawal reflex?

It is well established that, although the gross patterning of the somatosensory cortex may occur independently of activity, refinement relies on activity in the sensorimotor system (Pallas, 2001; Price et al., 2006; Inan and Crair, 2007; Hanganu-Opatz, 2010; Kolb et al., 2012; Blumberg, 2015; Cirelli and Tononi, 2015). In particular, injuring or deafferenting a limb during the first few days of life in the rat results in a failure of the development of the corresponding sensory cortex, as well as changes in sensory regions of the remaining limbs (Wall and Cusick, 1986; Dawson and Killackey, 1987; Waters et al., 1990; Pearson et al., 1999). Transecting the sensory regions of the spinal cord confirms that this effect reflects loss of ascending sensory, particularly proprioceptive, inputs (Jain et al., 2003). While it is hard to link this process to a particular pattern of brain activity, the altered development of the sensory cortex is only seen if the sensory feedback is disrupted during the first week of life, which overlaps with the peak period of expression of muscle twitches and spindle bursts (Blumberg et al., 2015).

The role of ascending sensory information depends on functional NMDA receptors in the cortex (Iwasato et al., 2000), suggesting it acts via Hebbian learning mechanisms as in the visual system. There is a critical period during the first week of life in which alterations in sensory feedback become reflected in the sensory representation at the cortex (Jeanmonod et al., 1981; Simons et al., 1984; McCasland et al., 1992). Do these adaptive changes reflect a role of spindle bursts in the sensory cortex? There is indirect evidence that they do. The normal development (Fox et al., 1996; Iwasato et al., 2000; Dagnew et al., 2003; Lee et al., 2005) of the barrel cortex, as well as the plastic responses following whisker removal (Schlaggar et al., 1993), depends on glutamatergic transmission via NMDA receptors. A second requirement for the normal barrel pattern is the intact subplate (Tolner et al., 2012). These same factors are necessary for the expression of spindle bursts (Minlebaev et al., 2008; Tolner et al., 2012). There is also indirect evidence from the visual system that spindle bursts may guide development. Spindle bursts in the visual cortex result from ascending sensory activity from spontaneous waves of retinal activity (Hanganu et al., 2006). As previously mentioned, it is well recognized that retinal waves are essential to the normal development of the visual system (Wong, 1999; Firth et al., 2005; Torborg et al., 2005). Much of this research has focused on the impact of retinal activity on retino-thalamic maturation, but it is clear that it impacts primary and even secondary cortical regions as well (Ackman et al., 2012). In particular, blocking retinal waves and presumably the subsequent cortical spindle bursts during the first week of life prevents normal ocular dominance column and receptive field formation in ferrets (Huberman et al., 2006; Alme et al., 2010) as well as normal precise connections from thalamus to the visual cortex in mice (Cang et al., 2005). Ablation of the subplate, also necessary for spindle bursts, also prevents the normal development of the visual cortex (Ghosh and Shatz, 1994; Kanold et al., 2003). These changes are not reversed by subsequent normal visual inputs (Hooks and Chen, 2007), again implying a critical function for spindle-burst associated patterns of activity in early life.

In summary, cortical development depends on activity, which refines neural connections via NMDA-receptor mediated by Hebbian mechanisms. One source of this activity is via ascending sensory inputs, and the spindle bursts generated by these inputs also depend on NMDA receptors. An intriguing possibility that follows is that spindle bursts are an essential pattern of activity that underlies the prolific activity dependent plasticity of early life, perhaps by synchronizing related groups of pre- and post-synaptic neurons (Mohajerani and Cherubini, 2006; Mohajerani et al., 2007). Falling spindle burst activity with increasing age could contribute to the closure of pre-critical periods for cortical plasticity (Feldman, 2001; Hensch, 2004; Erzurumlu and Gaspar, 2012; Kolb et al., 2012) and the beginning of the critical period in which the sensory inputs tune up the existing circuits. Processes other than the decrease in spindle bursts are also likely to influence the decreasing plasticity of the system in question. For example, NMDA receptor subunit expression undergoes substantial changes during the first week of life (Monyer et al., 1994), to configurations which are less favorable to synaptic potentiation (Erisir and Harris, 2003). Nevertheless, it is reasonable to suggest that spindle bursts represent the network correlate of NMDA receptor mediated activity-dependent plasticity in the developing cortex.

## Conclusions

In this review article, we have discussed two forms of spontaneous cortical activity present during sleep within sensory systems. In the adult, SWA is a prominent pattern activity seen during sleep or light sedation. Previously, it was thought to be a global phenomenon that synchronizes large swathes or cortex and sub-cortical regions. However, as new ideas about its role in scaling synapses within local systems have emerged, evidence has concurrently been gathered to show SWA has localized and regional features that reflect underlying functional cortical circuits. In the developing brain, spindle bursts are the dominant feature of sleep activity. In contrast to the adult brain, they rely only minimally on intrinsic brain circuitry and depend on ascending sensory inputs, which themselves are spontaneously generated by the developing nervous system. The precise roles for spindle bursts remain unclear, but their contribution in synaptogenesis and circuit connectivity is unquestionable.

We do not, however, claim any particular functional links between these two forms of activity. In fact, the independence of these patterns is a key message regarding the spontaneous activity of the brain. We believe it is neither noise nor a wasteful consequence of brain structure; it is an useful activity, purposefully generated, and used for specific and definable purposes.

## Author Contributions

DAM and MHM wrote the manuscript, material in this manuscript was used in DAM’s PhD thesis who was supervised by THM. THM edited the manuscript.

## Conflict of Interest Statement

The authors declare that the research was conducted in the absence of any commercial or financial relationships that could be construed as a potential conflict of interest.
